# Understanding the Influence of the Work Environment on Family‐Centered Care Among Nurses in Neonatal Intensive Care Units

**DOI:** 10.1155/nrp/6142926

**Published:** 2026-05-25

**Authors:** Majd Jada’a, Ahmad Ayed

**Affiliations:** ^1^ Department of Nursing, Faculty of Medical Sciences, Al-Quds University, Jerusalem, East Jerusalem, State of Palestine, alquds.edu; ^2^ Faculty of Nursing, Arab American University, Jenin, State of Palestine, aauj.edu

**Keywords:** family-centered care, neonatal intensive care unit, nurse, Palestine, work environment

## Abstract

**Background:**

Family‐centered care (FCC) is a cornerstone of neonatal intensive care, but its consistent implementation depends on nurses’ ability to collaborate with families within demanding clinical settings. The purpose of the study was to examine the influence of the nursing practice environment on FCC among nurses working in governmental neonatal intensive care units (NICUs) in the West Bank, Palestine.

**Methods:**

A cross‐sectional study was conducted from February 1 to May 1, 2025 in 11 governmental NICUs across the West Bank. A convenience sample of 210 NICU nurses completed a self‐administered questionnaire including demographic/professional characteristics, the Practice Environment Scale of the Nursing Work Index (PES‐NWI), and the Family‐Centered Care Questionnaire–Revised (FCCQ‐R).

**Results:**

The overall practice environment was rated relatively high (3.7 ± 0.6). The highest PES‐NWI domain was nurse foundations for quality of care (3.8 ± 0.6), while collegial nurse–physician relations (3.6 ± 0.8) was the lowest. Overall FCC was moderate (2.5 ± 0.5), with “family as the constant in the child’s life” (2.8 ± 0.7) highest and parent‐to‐parent support (2.4 ± 0.7) lowest. In regression analysis, age and overall practice environment were entered into the model, and practice environment was the only significant predictor of FCC (*B* = 0.130 and *p* = 0.015).

**Conclusion:**

NICU nurses reported a generally favorable practice environment and a moderate level of FCC. A stronger practice environment predicted higher FCC, underscoring the importance of organizational conditions for strengthening FCC implementation in Palestinian governmental NICUs.

## 1. Introduction

Neonatal intensive care units (NICUs) are inherently challenging care environments because they manage preterm and critically ill newborns who often require complex, technology‐dependent, and prolonged treatment [1]. In addition to high clinical acuity, NICU care involves rapid decision‐making, invasive procedures, resuscitation, and at times end‐of‐life decisions, all of which increase the emotional and technical demands placed on healthcare professionals and families [2]. For parents, a newborn’s admission to the NICU is often distressing because the unfamiliar environment, intensive monitoring, and limited participation in care may leave them feeling powerless and disconnected from their infant [3]. Parents of preterm infants may also experience guilt, shame, stress, and self‐blame, which can interfere with bonding and confidence in caregiving [4]. These realities make it essential for NICU care models to support both newborn outcomes and family well‐being.

One important response to these challenges is family‐centered care (FCC). FCC is an approach to care in which healthcare professionals and families form a collaborative partnership based on respect, information sharing, participation, and shared decision‐making, while recognizing the family as a constant in the child’s life [5, 6]. In neonatal care, FCC aims not only to improve parents’ experiences but also to support newborn health through stronger parent–provider communication, more consistent family involvement, and care that is responsive to the infant’s developmental and emotional needs [7]. Evidence suggests that family‐centered interventions can improve satisfaction, parental coping, and quality of care and can also contribute to better child and family outcomes, including stronger bonding and improved family well‐being [8–10].

In NICUs, FCC is particularly relevant because newborns depend on parents for attachment, comfort, and ongoing developmental support. The core elements of FCC commonly described in the literature are respect and dignity, information sharing, participation, and collaboration [6]. In practice, these elements mean involving parents in care and decision‐making, tailoring care to each family’s values and circumstances, communicating clearly and consistently, and creating opportunities for parents to participate in their infant’s care to the extent they are able [11, 12]. Previous studies have shown that FCC can reduce healthcare use, improve parent satisfaction and quality of life, strengthen family–child relationships, and reduce parental stress [8, 13, 14]. However, implementing FCC in the NICU is often difficult because the model requires sustained communication, trust, teamwork, and organizational support within a high‐pressure clinical setting [15].

Nurses play a critical role in implementing FCC in NICUs because they are the healthcare professionals who interact most consistently with parents, coordinate daily care, and translate unit policies into bedside practice [1, 12]. Through their communication, support, and inclusion of parents in care activities, nurses help operationalize FCC principles in everyday practice. However, several barriers may limit nurses’ ability to implement FCC effectively, including high workload, staffing shortages, inadequate space and infrastructure, limited organizational support, and inconsistent understanding of FCC principles across the unit [7, 12, 16]. One of the most important barriers is the work environment in which nurses provide care [17].

The nursing practice environment refers to the organizational characteristics of the workplace that facilitate or constrain professional nursing practice [18]. It includes staffing and resource adequacy, leadership support, nurse participation in hospital affairs, collegial nurse–physician relations, and foundations for quality of care [19]. A supportive practice environment enables nurses to communicate effectively, collaborate with other professionals, and provide care to the full extent of their role, whereas poor work environments marked by inadequate staffing, weak management support, and ongoing stress may undermine care quality and contribute to burnout and turnover intentions [20, 21]. In relation to FCC, previous studies suggest that organizational conditions and the NICU environment can either facilitate or hinder staff–parent partnership and family‐centered practice [16, 22]. Likewise, studies in critical care and pediatric settings have shown that better care environments are associated with more patient‐ and family‐centered care practices [17, 23, 24].

In Palestine, NICU nurses work within a healthcare system affected by resource constraints, heavy workloads, and high nurse–patient ratios, all of which may influence their ability to implement FCC consistently [25]. Despite the relevance of FCC in neonatal care, limited research has examined how the nursing practice environment relates to FCC among NICU nurses, particularly in Palestine and similar resource‐limited settings. Most local studies have focused on other aspects of nursing performance rather than the organizational conditions that may support or hinder FCC. Therefore, the present study aimed to examine the influence of the nursing practice environment on FCC among nurses working in governmental NICUs in the West Bank, Palestine. By addressing this gap, the study may help guide strategies to strengthen FCC implementation in culturally and resource‐constrained neonatal settings.

## 2. Methods

### 2.1. Design and Setting

This study used a cross‐sectional design and was conducted from February 1 to May 1, 2025, in 11 governmental NICUs located in the West Bank, Palestine. These units provide specialized care for preterm and critically ill newborns and together contain approximately 150 incubators. The participating NICUs are characterized by high patient demand, limited staffing, and constrained material resources, meaning that nurses often deliver care in busy settings where time, personnel, and equipment must be carefully prioritized. Governmental hospitals were selected because they are major providers of neonatal care services in Palestine and have well‐established Level II and Level III NICU services.

### 2.2. Population and Sample Size

A convenience sample was recruited from 11 governmental NICUs. The target population consisted of 427 nurses working in Level II and Level III NICUs in these hospitals. Sample size was estimated using Raosoft software as a precision‐based calculation rather than a hypothesis‐testing calculation. Assuming a 95% confidence level and a 5% margin of error, the minimum required sample was 203 nurses. To compensate for possible nonresponse, the recruitment target was increased to 230. A total of 210 nurses participated, yielding a response rate of 91.3%. Because the final sample exceeded the minimum required sample, the study was considered to have achieved the planned minimum sample size although it did not reach the inflated target set to account for nonresponse.

### 2.3. Inclusion and Exclusion Criteria

Eligible participants were nurses who were currently employed in the NICUs of the selected governmental hospitals in the West Bank, Palestine, and who had at least 6 months of NICU experience to ensure adequate exposure to NICU practice and FCC. Nursing interns, students, and nurses working in units that did not provide intensive care for neonates were not included.

### 2.4. Study Instruments

The questionnaire included three sections.

#### 2.4.1. Section One: Demographic and Professional Characteristics

This section composed of age, gender, educational level, experience in nursing, work shift, patients cared for in last shift, and FCC education.

#### 2.4.2. Section Two: Nursing Practice Environment

The Practice Environment Scale of the Nursing Work Index (PES‐NWI), developed by Lake [18], was used to assess the nursing practice environment. The scale contains 31 items distributed across five subscales: nurses’ participation in hospital affairs, nurse manager ability/leadership/support, foundations for quality of care, staffing and resource adequacy, and collegial nurse–physician relations. Responses are rated on a 4‐point Likert scale from 1 (*strongly disagree*) to 4 (*strongly agree*), with higher scores indicating a more favorable practice environment [26]. The PES‐NWI has been widely used to examine workplace factors that support or hinder nurses’ ability to provide high‐quality care [26] and has been recognized by U.S. healthcare organizations as a benchmark tool for assessing and promoting practice excellence [27, 28]. The scale showed good internal consistency, with Cronbach’s alpha ranging from 0.71 to 0.84 [18], and it has shown acceptable validity and reliability in prior studies conducted with Palestinian nurses [23]. The questionnaire was administered in English; therefore, no translation procedure was required.

#### 2.4.3. Section Three: FCC

FCC was evaluated using the Family‐Centered Care Questionnaire‐Revised (FCCQ‐R), a 45‐item instrument originally developed by Bruce and Ritchie [29]. The FCCQ‐R measures nurses’ perceptions of FCC across nine domains, including viewing the family as a constant in the child’s life, parent–professional collaboration, acknowledging each family’s individuality, information sharing, developmental needs, parent‐to‐parent support, emotional and financial support for families, health system responsiveness to family needs, and emotional support for staff. Items are scored on a 5‐point Likert scale ranging from *strongly agree* to *strongly disagree*. Subscale and total scores are calculated by summing item responses, with higher scores indicating better integration of FCC in nursing practice [5]. The FCCQ‐R has shown strong reliability, with reported internal consistency around 0.90 [5], and Cronbach’s alpha values ranging from 0.91 to 0.94 for the Current and Necessary dimensions in previous studies [30–32]. The questionnaire was administered in English; therefore, no translation procedure was required.

The Cronbach’s alpha coefficient for the study was 0.93 for the practice environment scale and 0.90 for the FCC scale.

### 2.5. Validity and Reliability

Content validity was ensured through the review of the questionnaire by a panel of experts, comprising three experts in nursing education and medical fields. Additionally, the study included the conduct of a pilot study involving 20 NICU nurses, where the feasibility and reliability of the measurement tools were assessed. The Cronbach’s alpha value obtained in the pilot study was found to be 0.83 for the practice environment tool and 0.84 for the FCC tool. In addition, in order to minimize the risk of bias, the nurses who participated in the pilot study were kept out from the actual study. It was found that the time required by the participants in completing the questionnaire was about 20–30 min, which is acceptable.

### 2.6. Ethical Considerations

Ethical approval was obtained from the Institutional Review Board (IRB) at Al‐Quds University (ref. no. RESC/2025‐28). In addition, administrative permission to conduct the study was obtained from the participating hospitals before data collection. The researcher explained the purpose of the study to the nurses, who were informed of their right to withdraw at any time without consequences. Confidentiality was ensured by not collecting or disclosing participants’ names or any identifying information. Nurses who agreed to participate provided written informed consent, and participation was entirely voluntary.

### 2.7. Data Collection Procedure

After obtaining ethical approval and formal administrative permissions, the researcher visited the selected hospitals and coordinated with NICU head nurses. The study objectives were explained, and permission was obtained to access the units and lists of eligible nurses. Paper‐based questionnaires in English were distributed to participants. Sealed envelopes were provided to ensure privacy when returning completed surveys. The researcher was available at the facilities to collect the questionnaires at the end of the workday to ensure that they were not lost or misplaced. The data collection was done from February 1 to May 1, 2025.

### 2.8. Data Analysis

Data were entered and analyzed using SPSS Version 27.0. The dataset was complete with no missing values, and the variables were normally distributed. Descriptive statistics (frequencies, percentages, means, and standard deviations) were used to describe the sample characteristics and study variables. Independent *t*‐tests, one‐way ANOVA, and correlation analyses were conducted to assess group differences and examine relationships. FCC was treated as the dependent variable because the study was conceptually designed to examine whether organizational conditions in the nursing practice environment predict nurses’ implementation of FCC. Variables entered into the multiple linear regression model were age and overall practice environment, as these variables were significantly associated with FCC in the bivariate analysis. Statistical significance was determined at *p* < 0.05.

## 3. Results

The mean age of the participants was 29.5 ± 4.4 years. Slightly more than half of participants (115 [54.8%]) were male. Most respondents (144 [68.6%]) held a Bachelor’s degree. On average, participants reported 4.9 ± 3.5 years of nursing experience. The majority (184 [87.6%]) works rotating shifts. During their most recent shift, nurses reported caring for an average of 4.3 ± 0.9 patients. Finally, more than two‐thirds of the participants (146 [69.5%]) indicated they had received FCC education, as seen in Table 1.

**TABLE 1:** Demographic characteristics of the study sample.

**Variable**		***n* (%)**	***M* (SD)**

Age			29.5 ± 4.4

Gender	Female	95 (45.2%)	
	Male	115 (54.8%)	

Educational level	Diploma	24 (11.4%)	
	Bachelor	144 (68.6%)	
	Master and above	42 (20.0%)	

Experience in nursing			4.9 ± 3.5

Work shift	Rotating shift	184 (87.6%)	
	Day	26 (12.4%)	

Patients cared for in last shift			4.3 ± 0.9

FCC education	Yes	146 (69.5%)	
	No	64 (30.5%)	

The analysis revealed that the overall nursing practice environment was rated relatively high, with a mean score of 3.7 ± 0.6. Among its subscales, the highest mean was for nurse foundations for quality of care (3.8 ± 0.6), while the lowest was for collegial nurse–physician relations (3.6 ± 0.8). In contrast, overall FCC was rated moderate, with a mean score of 2.5 ± 0.5. The highest FCC subscale score was for the family as the constant in the child’s life (2.8 ± 0.7), whereas the lowest was parent‐to‐parent support (2.4 ± 0.7), as seen in Table 2.

**TABLE 2:** Mean scores of nursing practice environment and family‐centered care domains.

Variable	*M* ± SD
Total practice environment	3.7 ± 0.6
Staffing and resources adequacy	3.7 ± 0.7
Nurse foundations for quality of care	3.8 ± 0.6
Nurse participation in hospital affairs	3.7 ± 0.7
Nurse manager ability, leadership, and support	3.7 ± 0.8
Collegial nurse–physician relations	3.6 ± 0.8
Overall family‐centered care	2.5 ± 0.5
The family as the constant of the child’s life	2.8 ± 0.7
Emotional support for staff	2.5 ± 0.6
Design of health care system	2.5 ± 0.6
Parent and professional collaboration	2.4 ± 0.5
Sharing information with parents	2.4 ± 0.6
Emotional and financial support for families	2.5 ± 0.7
Parent‐to‐parent support	2.4 ± 0.7
Developmental needs	2.4 ± 0.6

The analysis revealed that FCC has a significant weak negative correlation with age (*r* = −0.139 and *p* = 0.044), indicating slightly lower FCC scores with increasing age. FCC was also positively correlated with the practice environment (*r* = 0.179 and *p* = 0.009), indicating that better practice environments were associated with higher FCC scores, as seen in Table 3.

**TABLE 3:** Bivariate analysis of FCC among the participants.

Variable		*N*	*M* (SD)	Statistical test	*p*. value
Age				*r* = −0.139^∗^	0.044

Experience in nursing				*r* = −0.125	0.070

Number of patients				*r* = 0.104	0.133

Practice environment				*r* = 0.179^∗∗^	0.009

Gender	Male	115	2.47 ± 0.46	*t* = 0.510	0.610
	Female	95	2.43 ± 0.48		

Educational level	Diploma	24	2.49 ± 0.58	*F* = 0.143	0.867
	Bachelor	144	2.45 ± 0.48		
	Master and above	42	2.43 ± 0.37		

Work shift	Day	26	2.53 ± 0.37	*t* = 0.914	0.362
	Rotating shifts	184	2.44 ± 0.48		

FCC education	Yes	146	2.44 ± 0.44	*t* = 0.510	0.610
	No	64	2.48 ± 0.52		

In the multiple linear regression model, the overall model was statistically significant but explained a small proportion of variance in FCC (*R* ^2^ = 0.047, adjusted *R* ^2^ = 0.038, *F* = 5.125, and *p* = 0.007). The analysis showed that practice environment was the only significant predictor of FCC. A higher practice environment score was associated with higher FCC (*B* = 0.130 and *p* = 0.015), as shown in Table 4.

**TABLE 4:** Predictors of family‐centered care: multiple linear regression.

Family‐centered care								
Predictor	*B*	Beta	*t*	*p* value	95% CI		Collinearity statistics	
					Lower	Upper	Tolerance	VIF
Age	−0.013	−0.124	−1.817	0.071	−0.027	0.001	0.992	1.008
Practice environment	0.130	0.168	2.460	0.015	0.026	0.234	0.992	1.008
*R* ^2^ = 0.047, adjusted *R* ^2^ = 0.038, *F* = 5.125, *p* = 0.007, Durbin–Watson = 1.064								

## 4. Discussion

The current study indicated that nurses perceived their practice environment as highly favorable. The relatively high overall PES‐NWI mean suggests that, despite working in resource‐constrained governmental hospitals, NICU nurses in the West Bank perceived important supportive features in their workplaces. This interpretation is broadly consistent with ICU evidence from other countries showing positive overall practice‐environment ratings although absolute scores vary by setting, staffing patterns, and health‐system resources [33]. Viewed in context, these findings suggest that even under limited‐resource conditions, supportive professional relationships, leadership, and clinical standards can still be present and may help sustain care quality in NICUs.

Among the PES‐NWI domains, Nurse Foundations for Quality of Care received the highest score. This pattern is consistent with the literature, including the international benchmark meta‐analysis reporting that this subscale was often among the highest‐rated domains, particularly in hospital nursing settings [34]. In NICUs, this may reflect the strong emphasis on protocols, vigilance, technical competence, and safety that accompanies the care of critically ill neonates.

In contrast, the Turkish study reported lower scores for this domain [35]. One possible explanation is that differences in organizational structure, staffing adequacy, managerial support, and local professional norms may shape how nurses perceive quality foundations in their units. Differences in the hospital type and the specific clinical areas studied may also partly explain why the Turkish findings differed from the current NICU‐based results.

Other PES‐NWI domain scores in the current study were lower than Nurse Foundations for Quality of Care, suggesting that nurses viewed some elements of the work environment less positively than the professional and quality‐related aspects of care. This pattern may indicate that nurses were more confident in the clinical values and standards guiding care than in the organizational conditions needed to support those standards consistently in daily practice.

Collegial Nurse–Physician Relations was the lowest‐rated PES‐NWI domain in the present study. This finding is important because NICU care depends heavily on close interdisciplinary coordination. A lower score in this domain may reflect communication barriers, hierarchical decision‐making, or limited shared participation in care planning, all of which can constrain nurses’ ability to advocate for families and practice FCC consistently.

This ranking is similar to findings from Ethiopia, where nurse–physician collaboration was also the lowest‐rated domain [36]. However, it differs from the international benchmark meta‐analysis, which found that Nurse–Physician Collegial Relations was often one of the more highly rated domains in critical care settings [34]. These differences suggest that the relative ranking of PES‐NWI subscales is context dependent and may vary according to institutional culture, interdisciplinary norms, and local workforce pressures.

The overall FCC score in the current study was moderate. This suggests that FCC was present but not implemented at an optimal level in everyday NICU practice. Similar findings have been reported in pediatric and neonatal settings in other countries. For example, Coyne et al. [30], studying nurses in Ireland, reported a gap between the endorsement of FCC principles and their implementation in practice. Likewise, Dall’Oglio et al. [32], in Italian NICUs, identified significant differences between current and necessary FCC dimensions, indicating that staff perceived FCC as important but not yet fully achieved in practice.

This moderate overall FCC score is understandable in light of the Palestinian governmental NICU context. FCC requires time, communication, space for families, and team coordination. In busy public NICUs with high workloads and resource limitations, nurses may strongly support FCC in principle but still face practical barriers to translating that perspective into consistent daily behaviors.

At the same time, the relatively high score for “the family as the constant in the child’s life” appears at first to contrast with the moderate overall FCC score. This apparent contradiction likely reflects the difference between endorsing a core FCC belief and fully operationalizing all FCC dimensions. In other words, nurses may clearly value the family’s central role while finding it more difficult to implement behaviors such as shared decision‐making, structured information exchange, and parent‐to‐parent support under real clinical constraints.

The FCC domain pattern supports this interpretation. The highest domain was “the family as the constant in the child’s life,” indicating strong recognition of the family’s importance, which is in line with FCC principles of respect and dignity and with findings reported by Coyne et al. [30]. In contrast, parent‐to‐parent support was the lowest‐rated domain, while the remaining FCC domains clustered in a moderate range. This pattern suggests that attitudinal endorsement of family importance may be stronger than implementation of the more organizationally demanding dimensions of FCC.

Compared with other studies, this domain ranking is not fully consistent across settings. The Italian multicenter NICU study found higher practice scores for encouraging parent‐to‐parent support than for several other FCC domains [32], whereas Nusaifa and Joseph [37], in pediatric contexts, emphasized other FCC components such as recognition of family individuality, information sharing, and emotional support. These differences again suggest that the relative prominence of FCC dimensions may vary by country, unit type, and the extent to which FCC is embedded in routine service structures.

An important finding in the current study is that many respondents had previously received FCC training, yet the overall FCC score remained moderate. This suggests that knowledge alone may not be sufficient for implementation. Training may improve awareness of FCC concepts, but translating that knowledge into practice also depends on time, staffing, leadership, and institutional routines that enable nurses to involve families consistently.

The literature on FCC implementation is mixed. Some studies report stronger implementation where units have more structured support for FCC, clearer communication practices, and an organizational culture that actively promotes partnership with families. Other studies report lower implementation when workload is heavy, staffing is insufficient, or FCC remains more of an ideal than a routine operational model [30, 32, 38]. Presenting the evidence in this way helps explain why FCC implementation may vary substantially across countries and clinical units.

In the current study, the moderate FCC score can, therefore, be interpreted as partial implementation rather than absence of FCC. Nurses appear to recognize FCC as necessary and valuable, but not all dimensions are enacted to the same degree in practice. For clarity, the terms “current” and “necessary” FCC refer to the distinction used in the FCC literature between what staff perceive is presently occurring in clinical care and what they believe should ideally occur. Because the present study focused on perceived levels of FCC, these concepts are useful for interpretation even though they were not the primary analytic comparison in this study.

The findings related to levels of FCC should be considered before wider discussion of implementation. Overall, nurses perceived FCC at a moderate level rather than a high one. Studies from Ireland, Italy, and the United States have shown both similar and higher levels, depending on the instrument version used, the professional groups included, and whether the study was conducted in pediatric wards, ICUs, or NICUs [30, 32, 38]. The differences may reflect variation in institutional commitment to FCC, available space and resources for families, and the degree to which parents are integrated into routine care processes.

The central finding of this study is that the nursing practice environment significantly predicted FCC. In this manuscript, FCC was modeled as the outcome because the conceptual framework viewed the practice environment as an organizational condition that may support or constrain FCC. However, the relationship may also operate in the opposite direction or be reciprocal. Units in which nurses are more family focused may also cultivate more supportive teamwork and communication. Therefore, the current findings should be interpreted as evidence of association, not one‐way causation.

The regression finding suggests that organizational and clinical setting factors, rather than personal characteristics alone, are important in understanding FCC. Clinical setting should be understood here primarily as an organizational context because it reflects the structure, routines, resources, and interprofessional culture within which nurses provide care. This interpretation is supported by NICU‐focused evidence syntheses and related quantitative studies showing that the care environment can either enable or hinder staff–parent partnership and FCC practice [17, 22] although some studies also identify nurse competency and experience as important contributors [37, 39].

### 4.1. Strengths and Limitations

This study has several strengths. It included nurses from 11 governmental NICUs across the West Bank, which increases the relevance of the findings to public‐sector neonatal care in Palestine. The study also achieved a high response rate, reducing the likelihood of substantial nonresponse bias. In addition, the use of well‐established and validated instruments, both of which demonstrated excellent internal consistency in the current sample, strengthens confidence in the reliability of the measurements, and supports the credibility of the findings.

Several limitations should also be considered. The cross‐sectional design prevents causal conclusions, and the association between the practice environment and FCC may be reciprocal rather than unidirectional. The use of convenience sampling may limit generalizability beyond governmental hospitals in the West Bank. The data were based on self‐reported questionnaires and may, therefore, not fully reflect observed FCC behaviors. In addition, although the regression model was statistically significant, it explained a limited proportion of variance in FCC, indicating that other factors, such as NICU policies, family space, institutional culture, workload intensity, burnout, and psychosocial support resources, may also influence FCC practice.

### 4.2. Recommendations

Strengthening FCC in Palestinian governmental NICUs should prioritize improving the practice environment, not only individual nurse factors. Nurse managers and hospital leaders should enhance leadership support, strengthen nurse–physician collaboration, and standardize communication with families through strategies such as interdisciplinary rounds, shared care planning, and regular family updates. FCC education should be provided as ongoing, competency‐based training supported by supervision and clear unit standards. Because parent‐to‐parent support was the weakest FCC domain, NICUs should consider feasible peer‐support approaches adapted to staffing and space limitations. At the policy level, the Ministry of Health should develop NICU‐specific FCC guidelines that are integrated into staffing and quality‐improvement plans.

Future research should examine FCC from the perspectives of parents as well as nurses, use multicenter designs that include private and nongovernmental hospitals, and test interventions aimed at improving both the work environment and FCC. Studies exploring correlations between PES‐NWI and FCC subscales would also add depth by clarifying which dimensions of the practice environment are most closely linked to specific aspects of FCC.

## 5. Conclusion

NICU nurses in governmental hospitals in the West Bank reported high perceptions of their practice environment, whereas FCC remained at a moderate level. The nursing practice environment was significantly associated with FCC; however, it explained only a small proportion of the variance, indicating that additional factors beyond organizational conditions likely influence FCC. These findings suggest that while the practice environment contributes to FCC, it should be considered alongside other structural, interpersonal, and contextual factors when developing strategies to strengthen FCC in Palestinian NICUs.

## Author Contributions

The study was designed by Ahmad Ayed and Majd Jada’a.

The data was gathered by Majd Jada’a.

The data was analyzed by Ahmad Ayed.

## Funding

This research did not receive any specific grant from funding agencies in the public, commercial, or not‐for‐profit sectors.

## Disclosure

All authors prepared and approved the final version for submission. They agree to be accountable for all aspects of the work, ensuring accuracy and integrity.

## Ethics Statement

Ethical approval for this study was obtained from Al‐Quds University (RESC/2025‐28). Written informed consent was obtained from all subjects before the study. Participants were informed of their right to with draw at any time. All data were anonymized and stored securely.

## Consent

We affirm that this work is original and has not been published elsewhere, except as noted in the manuscript.

## Conflicts of Interest

The authors declare no conflicts of interest.

## Data Availability

The data that support the findings of this study are available on request from the corresponding author. The data are not publicly available due to privacy or ethical restrictions.
